# Introducing a novel low energy gamma ray shield utilizing Polycarbonate Bismuth Oxide composite

**DOI:** 10.1038/s41598-021-89773-5

**Published:** 2021-05-19

**Authors:** Rojin Mehrara, Shahryar Malekie, Seyed Mohsen Saleh Kotahi, Sedigheh Kashian

**Affiliations:** 1 Physics Department, K. N. Toosi University of Technology, Tehran, Iran; 2 Radiation Application Research School, Nuclear Science and Technology Research Institute (NSTRI), Tehran, Iran

**Keywords:** Materials science, Nanoscience and technology

## Abstract

The fabrication of different weight percentages of Polycarbonate-Bismuth Oxide composite (PC-Bi_2_O_3_), namely 0, 5, 10, 20, 30, 40, and 50 wt%, was done via the mixed-solution method. The dispersion state of the inclusions into the polymeric matrix was studied through XRD and SEM analyses. Also, TGA and DTA analyses were carried out to investigate the thermal properties of the samples. Results showed that increasing the amount of Bi_2_O_3_ into the polymer matrix shifted the glass transition temperature of the composites towards the lower temperatures. Then, the amount of mass attenuation coefficients of the samples were measured using a CsI(Tl) detector for different gamma rays of ^241^Am, ^57^Co, ^99m^Tc, and ^133^Ba radioactive sources. It was obtained that increasing the concentration of the Bi_2_O_3_ fillers in the polycarbonate matrix resulted in increasing the attenuation coefficients of the composites significantly. The attenuation coefficient was enhanced twenty-three times for 50 wt% composite in 59 keV energy, comparing to the pure polycarbonate.

## Introduction

X and γ-rays have a wide range of applications in military, medical, health, scientific, and agricultural industries. Increasing the utilization of hazardous radiations, including gamma sources in hospitals and research centers for diagnostic and therapeutic applications, has provided a much more unsecured place for personnel. So there will be a need to design an appropriate shield, depends on the type of ionizing radiation, to reduce the radiation dose in the intended site^1^. Study of interaction of radiation with matter is required to design a proper shield. The linear attenuation coefficient is the quantity to demonstrate the penetration of gamma-ray in the matter^2^. Since for high-Z elements, the photoelectric effect is dominant, heavy metals such as iron (Fe), lead (Pb), tungsten (W), with considerable density, have been widely used to attenuate gamma radiations^2^. Lead is toxic, chemically unstable, and heavy; therefore, researchers focused on alternative lead-free materials, which, besides being non-toxic, light-weighted, and flexible, should include the shielding properties of lead^3^.

Over the last years, polymer nanocomposites have been considered as novel materials in many fields, owing to their outstanding special physical and mechanical features by adding only a small amount of the nano reinforcements^4^. Polymer composites filled with nanoscale metal oxides are good candidates for shielding the gamma radiations, especially for diagnostic X‐rays below 150 kV^5^.

Generally, composites are containing a matrix part and a reinforcement phase. Polymers are light weighted, non-toxic, and flexible materials. The chief advantage of using polymers is their ability to be processed quickly, and they are excellent chemical resistance^6^. However, as they have low atomic numbers, they cannot be used as gamma radiation shields. Mostly a compounding agent with high-Z and high density is added as a reinforcement phase to enhance the gamma attenuation. Polymer composites have hydrogen-rich organic polymeric material as the matrix, which has an effective role in absorbing the neutrons when using as the shields. As a result of this property, the secondary radiations like Bremsstrahlung, produced in the presence of high-Z metallic shields such as Pb or Bi is reduced. In addition, it reduces the effective weight of the shielding material^7^. There is attention in the effects of the presence of nanoparticles in shielding materials due to their novel utilizes^8^. Two main factors that bring about different behaviors in the nano and micro particles are quantum effects and an increase in the surface to volume ratio factor in nanoparticles; these parameters affect the mechanical, thermal, and probably shielding properties of the material^9^. It is well-known that the dispersion of nanoparticles into the polymer matrix is much more challenging than micro-sized particles due to high surface area induced agglomeration^10^. According to the prominent features of nanoscale particles than micro-particles, it is causing an impressive increase in the attenuation coefficient. Studies show that combining the two phases of matrix and reinforcement is complex and different than each phase separately^11^. Polymers that have amorphous structures, like polycarbonate, PMMA, polystyrene, are better choices for making homogeneous nanocomposites. Several experimental results indicated that higher degree of polymeric matrix crystallinity hindered nanoparticle dispersion at higher weight percentages^12^. Polycarbonate is a thermoset polymer with features as being amorphous and recyclable^13^.

High-Z materials that have been considered as alternatives for lead and have nearly the same effects on shielding gamma radiations are mainly high density with high atomic numbers, such as tungsten oxide, bismuth oxide, barite^14,15^. As shown in Table 1, among these high-Z materials, Bi_2_O_3_ has a higher density, namely 8.9 g/cm^3^. Although the lead density is higher than bismuth oxide, being non-toxic is a much more critical issue.

**Table 1:** Properties of common materials for radiation shielding purpose.

Material	Density (g/cm^3^)	Linear attenuation coefficient for 200 keV gamma-rays	Nature
Lead	11.34	0.992	Toxic
Bismuth oxide	8.90	0.933	Nontoxic
Tungsten oxide	7.16	0.647	Non-toxic
barite	4.48	0.288	Non-toxic

Until now, different researchers have studied lead-free composite shields. El-khatib et al. designed and fabricated composites consist of polyethylene (HDPE) mixed with micro-sized and nano-sized cadmium oxide (CdO) particles for attenuation of gamma rays with energy ranging from 59.53 keV up to 1408.01 keV. It was obtained that nanoscale reinforcement enhanced the shielding properties, especially at lower photon energies^16^. The authors have investigated tungsten oxide-polymer composite theoretically by the Monte Carlo method for various gamma energies from 50 keV to 1.33 MeV. The linear attenuation coefficients by nanostructured and microstructured of 50 wt% WO_3_/E44 epoxy composites were compared. The results showed that WO_3_ nanoparticles tend to increase the linearattenuation coefficient in comparison with microparticles^17^. Kazemi et al. studied new polyvinyl alcohol (PVA)/WO_3_ composite in the presence of high-energy gamma photons through simulation via the Monte Carlo N-Particle (MCNP) simulation code. They found that the PVA/WO_3_ composite can be considered as a shield for the gamma energy at the level of 662, 778, 964, 1112, 1170, 1130, and 1407 keV^18^. Later, Atashi et al. fabricated a flexible silicone rubber/W/Bi_2_O_3_ by an open mold cast technique^19^. Final composites result in a high attenuation coefficient for gamma rays. Besides, it has been shown that by increasing Bi_2_O_3_ in composites, the agglomeration of fillers decreases. In another work by Gavrish et al., an improvement in thermophysical, radiation-shielding, and mechanical properties was found by varying the amount of tungsten nanopowder^20^. Bi_2_O_3_ was dispersed in Bi_2_O_3_/XNBR films, in the concentration range of 30–70 wt% by Liao et al. These films had an effective role in attenuating low energy gamma rays^21^.

Another piece of work that deserves special mentions is a study of the gamma attenuation property of UHMWPE/Bi_2_O_3_ by Abdalsalam et al.^22^, in which samples were fabricated by adding 0.5, 1, 1.5, and 2 wt% of bismuth oxide into ultra-high molecular weight polyethylene and then using hot-press. The results of EDX analysis confirmed that by increasing the amount of bismuth in the composite, an increase of the peak related to bismuth was observed, which indicated the homogeneity and availability of bismuth in the composite. Also, they evaluated the presence of the bismuth oxide nanoparticles in the composite using XRD analysis via the determination of the obtained peaks and their corresponding crystal planes. Raman spectroscopy is a significant tool to study the molecular structure in metal oxide^22^. Results of the study indicated that the presence of the small amounts of bismuth would not change the Raman spectrum. Finally, measurements of $(\frac{\mu }{\rho })$ for energies between 30.8 and 383.9 keV showed that the sample with 2 wt% Bi_2_O_3_ exhibited the highest photon attenuation.

Studies by Verdipoor et al. on silicon resin and WO_3_, PbO, and Bi_2_O_3_ micro and nanoparticles, as reinforcement, showed that depending on filler concentration, the nanoparticles had higher mass attenuation coefficients, in which ^60^Co, ^137^Cs, and ^133^Ba sources were used to investigate radiation shielding properties^23^. According to work done by Ambika et al., results of using 60 wt% filled polymer composite as a shield against low gamma energies are comparable to that of barite. It also proved to be lightweight in comparison with conventional shielding materials^24^.

In this paper, polycarbonate/Bi_2_O_3_ nanocomposites with different weight fractions of nano Bi_2_O_3_ were prepared using the mixed-solution method. Then the samples were molded by a hot press in order to prepare samples with uniform thicknesses. For each sample, the mass attenuation coefficient was measured, using low energy gamma sources of ^241^Am, ^57^Co, ^99m^Tc, and ^133^Ba. Also, SEM tests were done to study the amount of agglomeration that occurred in each sample. Later XRD and TGA tests were carried out on each sample.

## Materials and methods

Reinforcements adding to the polymer matrix initially will distribute all over the polymer. However, by starting crystallization, they will be refused by crystal parts and indwell in amorphous parts of the polymer^10^. That is why it is crucial to select a polymer that its structure is mostly amorphous than a crystallite. Amorphous tend to have better dimensional stability and impact resistance^25^. Therefore In this research, polycarbonate with a density of 1.2 g/cm^3^ has been chosen as the matrix of the nanocomposite. Polycarbonate has an amorphous structure that can distribute nanoparticles uniformly. To impart radio-protective properties, Nano Bi_2_O_3_, as a non-toxic heavy metal oxide with 8.9 g/cm^3^ density and an atomic number of 83, was chosen as the reinforcement of the nanocomposite. Bi_2_O_3_ is a direct bandgap semiconductor. A characteristic feature of Bi_2_O_3_ consists of its polymorphism: five modifications, known as α-, β-, γ-, δ-, and ω-Bi_2_O3, were outlined^26^. This refractory high-Z material has a melting point of 817 °C and available in powder form^3^.

Toward fabricating polycarbonate/Bi_2_O_3_ nanocomposite samples, PC C-206 polycarbonate granule with a repeat unit of molecular weight of 254.3 g/mol and an MFI of 7.1–10 g/10 min (300 °C) and size of 2 mm was provided from the Iranian-Khouzestan petrochemical company. Bi_2_O_3_ nanopowder with a particle size of 90–210 nm and 99.8% trace metals basis was purchased from Sigma-Aldrich.

### Fabricating the nanocomposite

The preparation of nanocomposites is known to be a challenging task due to the high surface energy of the nanoparticles. Surface interactions between nanoparticles and polymer matrix have a significant efficacy on the properties of the final product^27^. Non-homogeneous distribution of nanoparticles in the matrix part and agglomeration of particles in nanocomposites increases the surface energy, which may cause the debilitation of nanocomposite structure, so choosing a proper way to manufacture the pieces is essential^10^. Due to these facts, polycarbonate and Bi_2_O_3_ nanopowder were mixed by solution processing. Polycarbonate has a phenyl group in its structure; thus, the chosen solvent should be able to break these chains, so the final product becomes more flexible. Dichloromethane, with a boiling point of 39.6 °C, was utilized as a solvent for polycarbonate. Mass of polycarbonate and Bi_2_O_3_ were calculated, as shown in Table 2, for 0, 5, 10, 20, 30, 40, and 50 weight percentages (wt%). Also, as can be seen from Table 2, the density of the composites was calculated and depicted.

**Table 2:** Measured amounts of PC and Bi_2_O_3_ to prepare nanocomposites.

Bi_2_O_3_ wt%	Mass of PC (g)	Mass of Bi_2_O_3_ (g)	Mass of nanocomposite (g)	Density (g/cm^3^)
0	8	0.00	8.000	1.15
5	8	0.421	8.421	1.19
10	8	0.889	8.889	1.24
20	8	2.000	10.00	1.36
30	8	3.429	11.429	1.54
40	8	5.333	13.333	1.74
50	8	8.000	16.000	1.97

Initially, 8 g of polycarbonate was dissolved in 40 ml dichloromethane on a magnetic stirrer-heater at 40 °C. Dichloromethane is quickly evaporated; hence a piece of aluminum foil was applied to cover the top of the beaker. After 45 min, the polycarbonate was fully solved in dichloromethane. Then the mixture of polycarbonate solution was loaded with different nano Bi_2_O_3_ levels. The temperature of the mixture was kept above the boiling point of dichloromethane, ensure that boiling helps the nanopowder to become dispersed all over the composition. In order to gain a better result, an ultrasonic bath was used, followed by the solution casting. At this stage, the composite was put into an ultrasonic bath for 10 min; this allows achievement of a uniform distribution of highly dispersed reinforcements in the polymer matrix for all weight fractions of 5, 10, 20, 30, 40, and 50 wt%, these steps were repeated. Final products were poured into a silicone mold to cool down at room temperature.

In order to measure the attenuation coefficient of the samples in the laboratory, they should have a specific and fixed thickness as well as a uniform surface. For achieving that aim, a hot press was used to mold the nanocomposites. Since dichloromethane molecules are still present in the structure, pressing them at high temperatures causes the additional solvent to trap as bubbles, which may cause additional problems during the experiments. So polycarbonate/Bi_2_O_3_ nanocomposites were put into the oven at 80 °C for 30 min and then were hot-pressed in a mold with dimensions of 8 × 8 × 0.1 cm^3^; thus polycarbonate starts melting at a rate of 220–240 °C temperature. In lower temperatures, the melting process will not be done correctly, and in higher temperatures, after cooling down, the polycarbonate will become brittle. So it is essential to work in the aforementioned range of temperatures. Samples were preheated at a temperature of 240 °C for 10 min and then were pressed at 240 °C and pressure of 200 atm for 1 min. Then pressed immediately via a cold press for 10 min at a pressure of 246 atm^26^. This process was continued for the other samples, namely 5, 10, 20, 30, 40, and 50 wt%.

### Determination of mass attenuation coefficient

The specimens were irradiated by gamma sources to investigate the radiation shielding properties of the nanocomposites based on PC, modified by Bi_2_O_3_. Each sample was cut into four pieces with dimension of 4 × 4 × 0.1 cm^3^ to measure $(\frac{\mu }{\rho })$ for different thicknesses of each filler level and to calculate half-value layers (HVLs). Even though mold was used for pressing the specimens, the thickness of the final pieces was not identical, so the thickness of the pieces was measured using a digital micrometer. Measurements were done by using a CsI(Tl) detector with 10% resolution on an energy peak of 662 keV of ^137^Cs source. The detector consists of a multichannel analyzer (MCA) and ^99m^Tc, ^241^Am, ^57^Co, and ^133^Ba as low energy gamma sources, according to Table 3. Each piece was located in front of the source, and the energy distribution of incident photons and transmitted gamma rays were recorded for a fixed preset time of 120 s. considering the short half-life of ^99m^Tc (6 h), before locating each piece, the intensity of the incident beam was measured as well.

**Table 3:** Gamma energy sources for measuring the mass attenuation coefficient.

Source	Gamma energy (keV)	Half-life
^241^Am	59	432.2 years
^57^Co	122	70.86 day
^99m^Tc	140	6 h
^133^Ba	356	10.51 years

Each sample was cut into four equal pieces, and measurements were repeated five times at different thicknesses. According to Eq. (1), the slope of the diagram of ln(*I*_*0*_/*I*) on *ρx*, determines the mass attenuation coefficient^28^.

1 $$\mathrm{I}\left(\mathrm{x}\right)={\mathrm{I}}_{0}{\mathrm{e}}^{- \left(\frac{\upmu }{\uprho}\right) \cdot \mathrm{\rho x}}$$

Thus, *I*_*0*_ is the photopeak area of the incident photons spectrum, and *I* is the photopeak area of the transmitted spectrum. In continue, half-value layer (HVL) and tenth value layer (TVL) were calculated using the following formulas:

2 $$\mathrm{HVL}=\frac{\mathrm{ln}2}{\upmu }$$

3 $$\mathrm{TVL}=\frac{\mathrm{ln}10}{\upmu }$$

**Figure 1:**
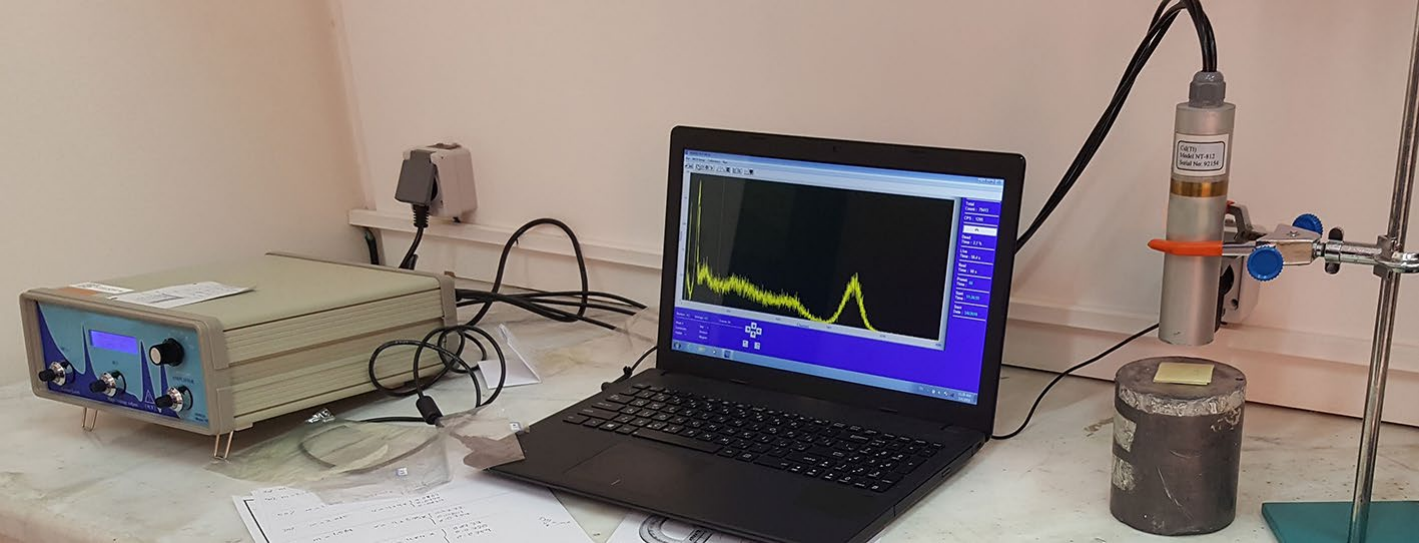
Set up of the experiment in the laboratory.

The experimental setup has been shown in Fig. 1.

Figure 1 shows the setup of measurement. The adjustment of the energy window was made by using a multichannel analyzer. The peak intensity of the gamma-rays was calculated as the peak area. During the experiments, the detector dead-time was under 4%. Afterward, considering the interaction of photons with matter, the results are discussed. In addition, the results of measurement were compared with Cadmium Oxide/high-density polyethylene nanocomposite^16^. The standard deviation for experimental data was calculated using the following equation:

4 $$\Delta \left(\frac{\mu }{\rho }\right)=\frac{1}{\rho t}\left(\frac{\Delta {I}_{0}}{{I}_{0}}+\frac{\Delta I}{I}+\frac{\Delta \rho }{\rho }\mathit{ln}\left(\frac{{I}_{0}}{I}\right)+\frac{\Delta x}{x}\mathit{ln}\left(\frac{{I}_{0}}{I}\right)\right)$$

Which ΔI_0_, Δ*I*, and Δ*ρ* are the standard deviation of measuring *I*_0_, *I* and *ρ* respectively^29^.

## Results and discussion

### SEM analysis

**Figure 2:**
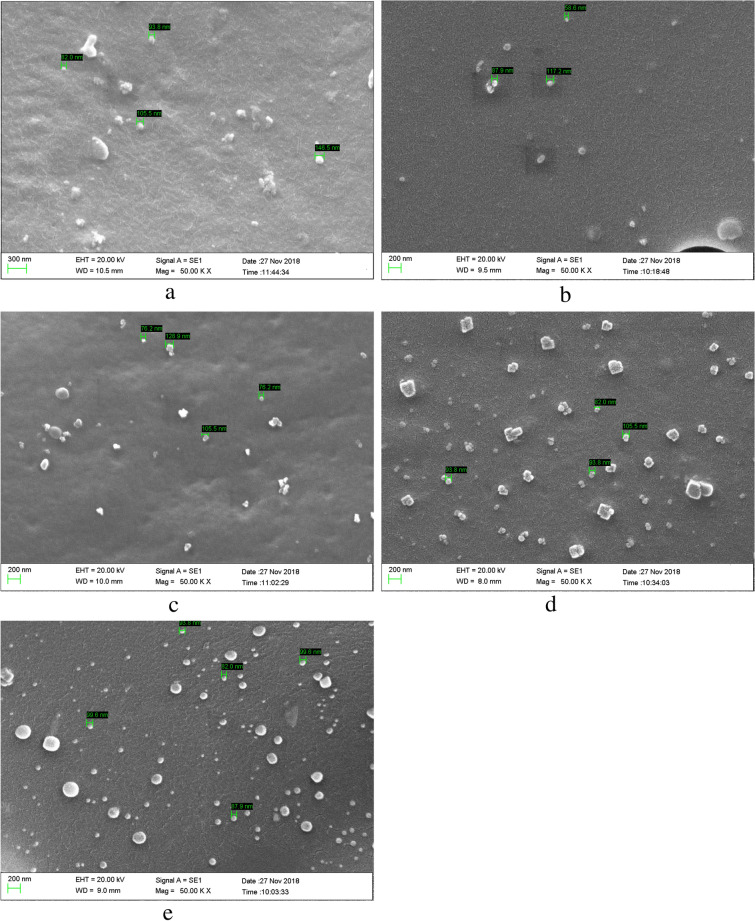
SEM images of the nanocomposite for different weight percentages of Bi_2_O_3_. (**a**) 5 wt%, (**b**) 10 wt%, (**c**) 30 wt%, (**d**) 40 wt%, and (**e**) 50 wt%.

Morphology tests were done on each sample using the scanning electron microscope, ZEISS EVO 10, to analyze the dispersion state of Bi_2_O_3_ in polycarbonate^3^. SEM tests show that the size of the nanoparticles is the same as the size of purchased nanoparticles (90–210 nm); As can be seen from Fig. 2, SEM images showed a uniform dispersion of the nano-fillers into the polymer matrix at the different Bi_2_O_3_ wt%.

SEM analyses proved that the dispersion quality of nanoparticles into polycarbonate varies based on the weight fraction. The chemical structure and physical properties of both the polymer and the nanoparticles are different. Although blending two components was done long enough to ensure the uniform dispersion of the Bi_2_O_3_ into polycarbonate, due to higher density and/or lack of interaction or bonding with polymer pellets, there are still possibilities that some particles have been settled down^30^. Results of tests showed that large agglomerations were not found through the polymer.

### XRD analysis

The phase of the formed metal oxide is determined from X-ray diffraction (XRD) analysis^31^. XRD data were collected on a PANalytical X´PertPro powder diffractometer (CuKα *λ* = 0.15496 nm), in order to study the structure of PC/Bi_2_O_3_ nanocomposites and changes of the structure from pure polycarbonate to 50 wt% PC/Bi_2_O_3_ nanocomposite. XRD analysis for all specimens was recorded. The records were carried out with the use of a graphite monochromator over the 2θ range of 5–40° with a pitch angle of 0.026*°* at room temperature. The generator was set on 40 mA and 40 kV. In order to estimate the crystallites size of Bi_2_O_3_ nanoparticles, the Scherrer equation was used^20^.

5 $$\mathrm{D}=\frac{\mathrm{k\lambda }}{\mathrm{\beta cos}(\uptheta )}$$

where *λ* is the wavelength of Cu kα in nm, *k* is equal to 0.9, *β* is the full width at half-maximum (FWHM) of the diffraction peak, *θ* is the diffraction angle and *D* is the average diameter of the particle in nm. For example, the size of the crystallites at 2*θ* values of 27.45° and 33.3° ,using Scherrer formula, evaluated as 76 and 78 nm.

**Figure 3:**
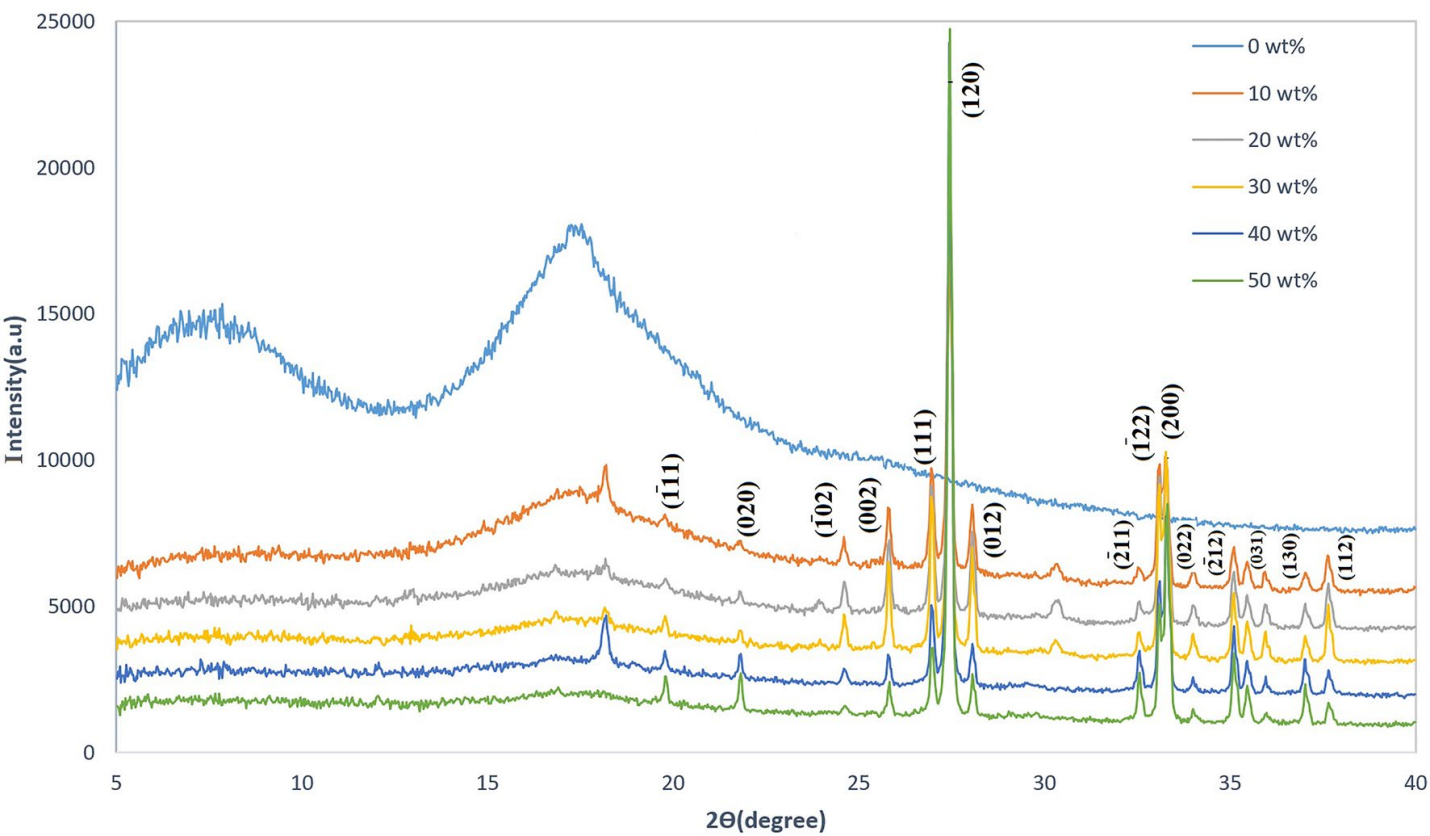
XRD spectra for different filler levels of PC/Bi_2_O_3_ nanocomposites.

**Figure 4:**
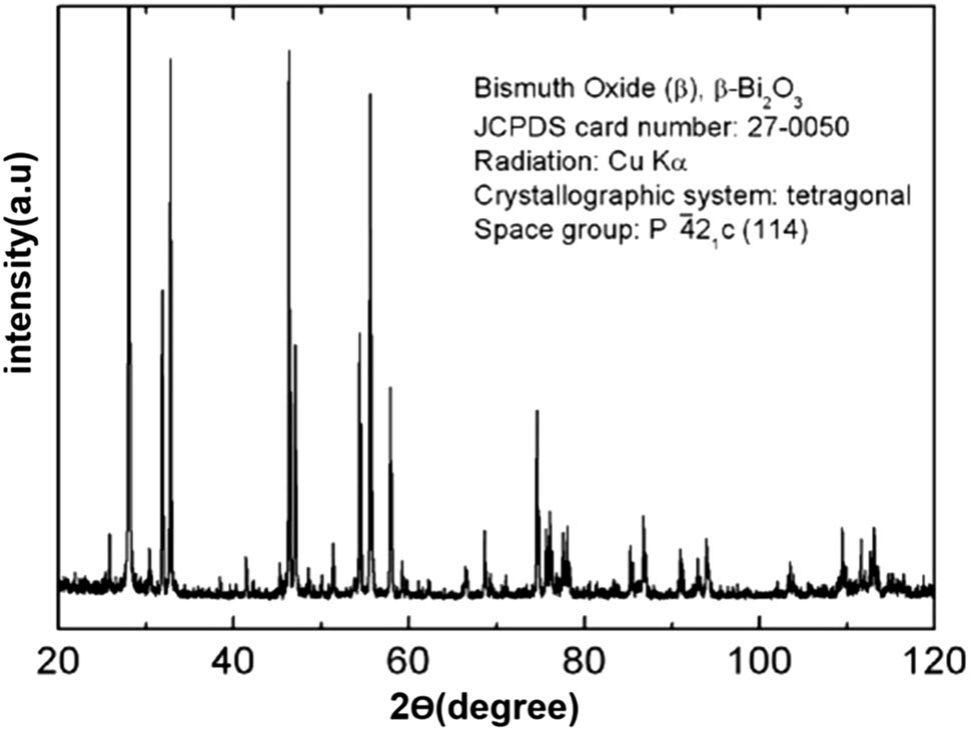
Reference XRD spectra of Bi_2_O_3_ nanopowder.

XRD spectra of pure polycarbonate and PC/Bi_2_O_3_ nanocomposites are shown in Fig. 3 We shall see that PC spectra have a 2*θ* = 16° broad peak, indicating an amorphous structure^32^. By adding Bi_2_O_3_, sharp peaks will appear until 50 wt% of the nano-fillers in which the peaks are rather sharp, and broad-peaks can hardly be seen. Also, by adding Bi_2_O_3_ nanopowder, the nanocomposite structure will have a path from amorphous to a crystalline structure. Sharp peaks reaffirm the semi-crystalline/crystalline structure of specimens^33^. As can be seen from Fig. 4, the obtained patterns were in good agreement with the standard JCPDS file number 76-1730, which corresponds to the monoclinic phase of Bi_2_O_3_^34^.

As can be seen from Fig. 3, the peaks at 2θ of 19.78º, 21.8º, 24.59º, 25.81º, 26.95º, 27.45º, 28.04º, 32.54º, 33.09º, 33.3º, 33.97º, 35.09º, 35.46º, 37.01º and 37.64º correspond to the ($\overline{1 }11$), ($020$), ($\overline{1 }02$), ($002$), ($111$), ($120$), ($012$), ($\overline{2 }11$), ($\overline{1 }22$), ($200$), ($022$), ($\overline{2 }12$), ($031$), ($130$) and ($112$) reflections of Bi_2_O_3_, in which exhibit a good agreement with the other researches^34,35^ .

According to obtained results, by increasing the concentration of Bi_2_O_3_ nanopowder into the polymer matrix, the nanocomposite structure becomes more crystalline. After the added Bi_2_O_3_ exceeded 50 wt%, the main peaks get sharper, and the FWHM of the peaks decreases, so that the XRD pattern of the PC/Bi_2_O_3_ nanocomposites is getting closer to that of Bi_2_O_3_ and the peak from polycarbonate is hardly distinguishable.

The details of crystal planes for 10 wt% PC/Bi_2_O_3_ nanocomposite are exhibited in Table 4 for 10 wt% PC/Bi_2_O_3_ nanocomposite.

**Table 4:** The details of crystal planes for 10 wt% PC/Bi_2_O_3_ nanocomposite.

Pos. [2θ]	Height [cts]	FWHM left [2θ]	d-spacing [Å]	Rel. int. [%]
17.17(3)	365(46)	1.1(2)	5.16138	3.56
18.150(4)	982(86)	0.14(2)	4.88369	9.58
19.76(1)	247(57)	0.12(3)	4.48975	2.41
21.77(1)	241(46)	0.22(7)	4.07943	2.35
24.58(2)	607(77)	0.12(4)	3.61832	5.92
25.782(8)	1588(94)	0.11(2)	3.45279	15.49
26.946(4)	2749(101)	0.11(1)	3.30619	26.8
27.419(2)	10,256(136)	0.116(4)	3.25026	100
28.035(6)	1832(82)	0.12(1)	3.18024	17.86
30.31(4)	381(39)	0.28(9)	2.94629	3.71
32.53(2)	446(60)	0.12(4)	2.75054	4.35
33.076(4)	3359(302)	0.111(9)	2.7061	32.76
33.263(3)	3681(143)	0.14(2)	2.69129	35.89
33.99(2)	441(48)	0.15(4)	2.63535	4.3
35.077(8)	1155(59)	0.15(2)	2.5562	11.26
35.440(6)	791(47)	0.14(2)	2.53083	7.71
35.93(1)	577(51)	0.09(2)	2.49768	5.62
37.01(2)	509(47)	0.15(4)	2.42718	4.96
37.625(7)	1072(53)	0.13(2)	2.38875	10.45

The crystal structure of Bi_2_O_3_ may not affect radiation attenuation below 10 wt%. Nevertheless, the introduction of a higher amount of Bi_2_O_3_ expands the crystal parts, which is essential for modifying the gamma radiation properties (for higher weight fractions of Bi_2_O_3_, the crystal parts expand, and photoelectric cross-section for the absorption of gamma-rays is increased which results in better radiation attenuating). Higher amounts of the inclusions, which lead to an increase in the density of the nanocomposites, increases the probability of the interaction of photons with matter.

### TGA and DTA analyzes

To study the influence of the mentioned filler on the heat resistance of the obtained composites, TGA analysis of nanocomposites with different levels of Bi_2_O_3_ was carried out. Thermal characteristics implemented in the range of 20–600 °C. The decomposition temperature obtained from TGA is a measurement of thermal stability^36^.

**Figure 5:**
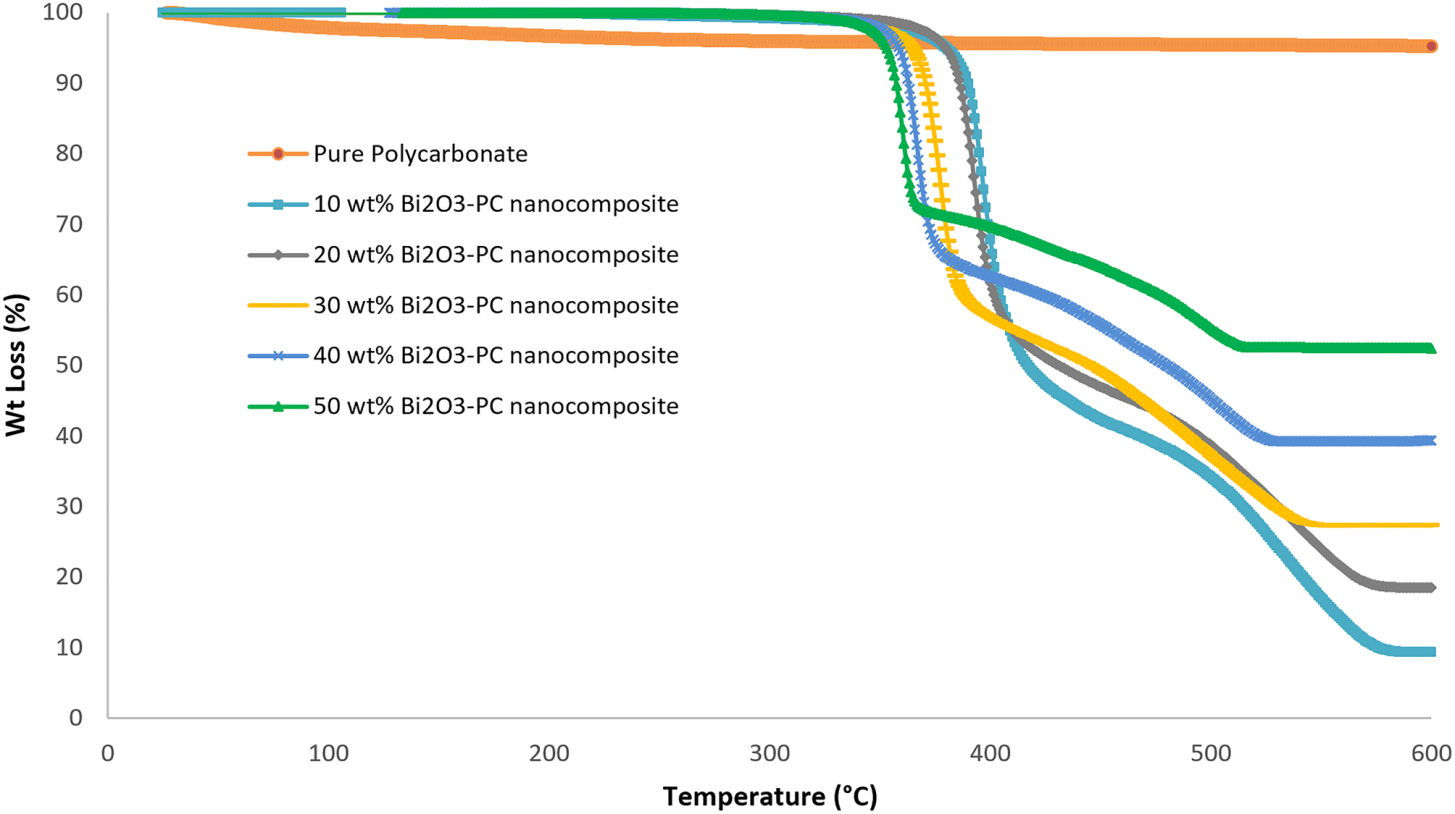
TGA thermograms of nanocomposites containing different levels of Bi_2_O_3_.

Thermogravimetric analysis (TGA) was carried out at air atmosphere and heating rate of 10 °C/min to investigate the thermal performance of the nanocomposites using Rheometric scientific STA 1500. Figure 5 shows TGA plots for different weight fractions of the PC-Bi_2_O_3_ nanocomposites_._ It is interesting to note that for pure PC, there is no weight loss till 600 °C, while other experimental works including Charde et al. showed that pure PC was degraded 50 wt% at 510 °C. Maybe, this is related to the fact that the kind of polymer, especially its grade, can exhibit different thermal behaviors of the polymer in TGA and DTA analyses. By adding 10 wt% of Bi_2_O_3_ to the matrix, thermal decomposition happens at 400  °C, and by increasing the filler level, it happens in lower temperatures. It can be concluded that increasing the Bi_2_O_3_ nanofillers into the polymer matrix may lower the glass transition temperature. Also, it can be mentioned that as the filler content increases, the rate of weight loss for the nanocomposites decreases^37^. The glass transition temperature of a polymer nanocomposite generally depends on the glass transition temperature of both the polymer and nano-filler materials and the weight fractions of both^38^.

**Figure 6:**
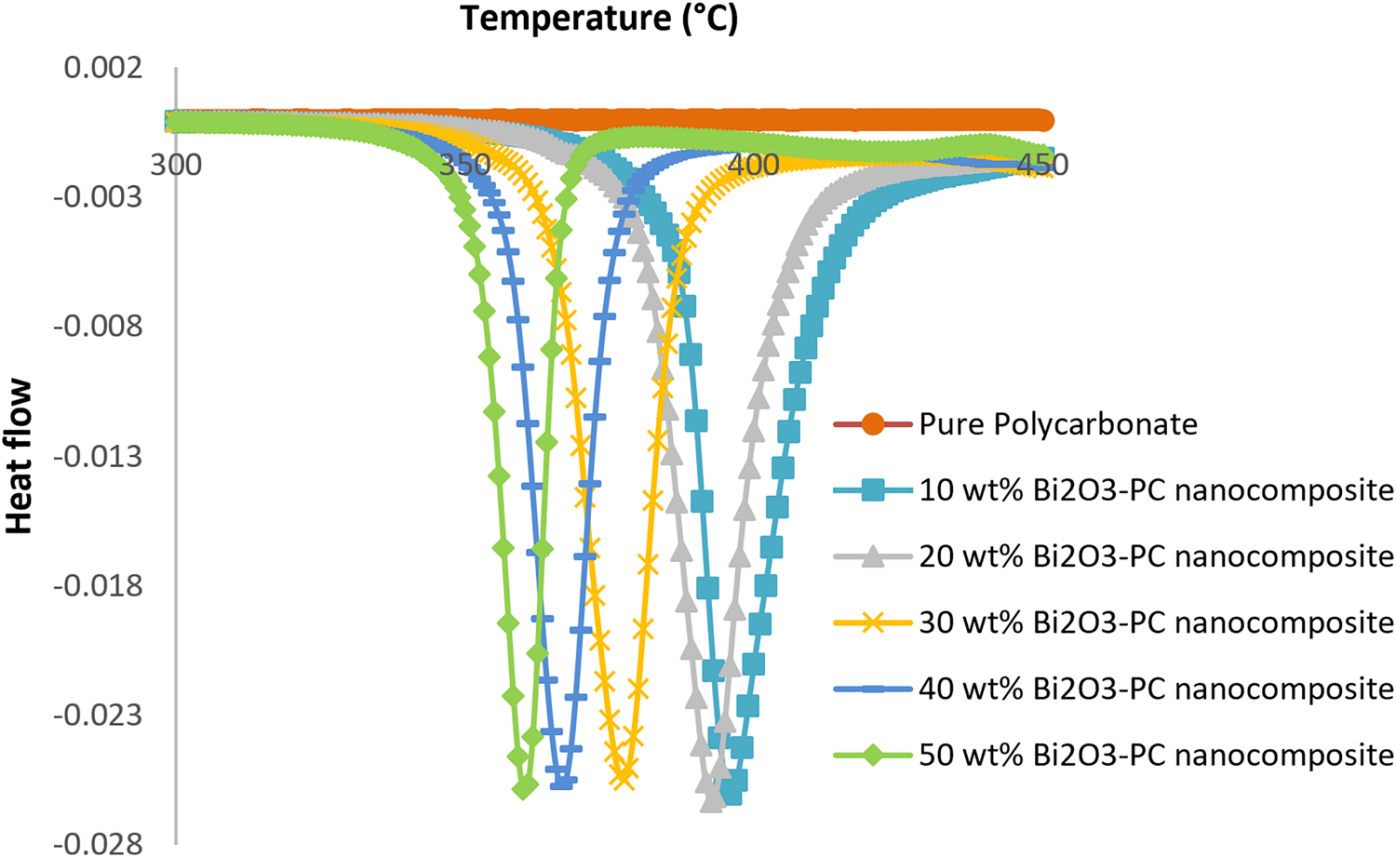
DTA thermograms of nanocomposites containing different levels of Bi_2_O_3_.

Differential Thermal Analysis (DTA) is a technique for identifying the thermal changes and reactions in the composites in a particular temperature range of 80–750 °C. DTA analysis can detect the discontinuous changes in specific heat, which are associated with such transitions^21^. Figure 6 and Table 5 reveal that, for the thermograms of the nanocomposites with low filler concentrations, the glass transition temperature of the nanocomposites shifts towards the lower temperatures. This phenomenon was also observed in the TGA analysis in Fig. 5. These results are in good agreement with previous works^26,39^. To justify this phenomenon, it can be mentioned that adding the amount of bismuth oxide nanoparticles into the polymer matrix leads to stronger Van der Waals forces between the nanoparticles; thus, the tendency to agglomeration is increased. Maybe this agglomeration reduces the crystallinity of the polymer nanocomposite, and significantly the amorphous region will be increased, therefore Tg will be reduced. Generally, more crystallinity in the polymer matrix leads to achieve higher Tg.

**Table 5:** DTA results for nanocomposites containing different levels of Bi_2_O_3_.

sample	T_g_ (°C)
10 wt% PC/Bi_2_O_3_ nanocomposite	393
20 wt% PC/Bi_2_O_3_ nanocomposite	392
30 wt% PC/Bi_2_O_3_ nanocomposite	378
40 wt% PC/Bi_2_O_3_ nanocomposite	365
50 wt% PC/Bi_2_O_3_ nanocomposite	360

### Radiation shielding properties

Measurements showed that incorporating a small amount of Bi_2_O_3_ nanopowder into the polycarbonate matrix makes negligible changes both in the attenuation of gamma radiation and structure. Determining the mass attenuation coefficient from the diagram of each measurement showed that the mentioned nanocomposites have an impressive effect on shielding low energy gamma radiations, as can be seen from the Table 6. A comparison with CdO/HDPE nanocomposites showed that in lower gamma energies, the mass attenuation coefficient of Bi_2_O_3_/PC nanocomposites is up to 1.5 times more than Nano CdO/HDPE. At higher energies of photon, the results showed almost higher mass attenuation coefficient for Nano CdO/HDPE.

**Table 6:** Mass attenuation coefficient results for each weight fraction and different energy sources.

Filler (wt%)	Gamma energy (keV)	$\frac{\mu }{\rho }({\mathrm{cm}}^{2}/\mathrm{g})$	
		Nano Bi_2_O_3_/PC	Nano CdO/HDPE^13^
0	59	0.12 ± 0.02	0.18
	122	0.14 ± 0.01	0.16
	140	0.12 ± 0.08	–
	356	0.07 ± 0.07	0.11
5	59	1.78 ± 0.21	–
	122	0.3 ± 0.02	–
	140	0.16 ± 0.07	–
	356	0.06 ± 0.03	–
10	59	1.91 ± 0.32	0.79
	122	0.37 ± 0.05	0.25
	140	0.23 ± 0.08	–
	356	0.07 ± 0.07	0.12
20	59	2.23 ± 0.28	1.37
	122	0.58 ± 0.13	0.32
	140	0.38 ± 0.08	–
	356	0.11 ± 0.01	0.12
30	59	2.28 ± 0.11	1.93
	122	1.03 ± 0.01	0.39
	140	0.58 ± 0.11	–
	356	0.10 ± 0.01	0.13
40	59	2.73 ± 0.12	2.56
	122	1.33 ± 0.12	0.46
	140	0.61 ± 0.08	–
	356	0.11 ± 0.02	0.13
50	59	2.93 ± 0.41	–
	122	1.64 ± 0.01	–
	140	0.67 ± 0.06	–
	356	0.13 ± 0.01	–

**Figure 7:**
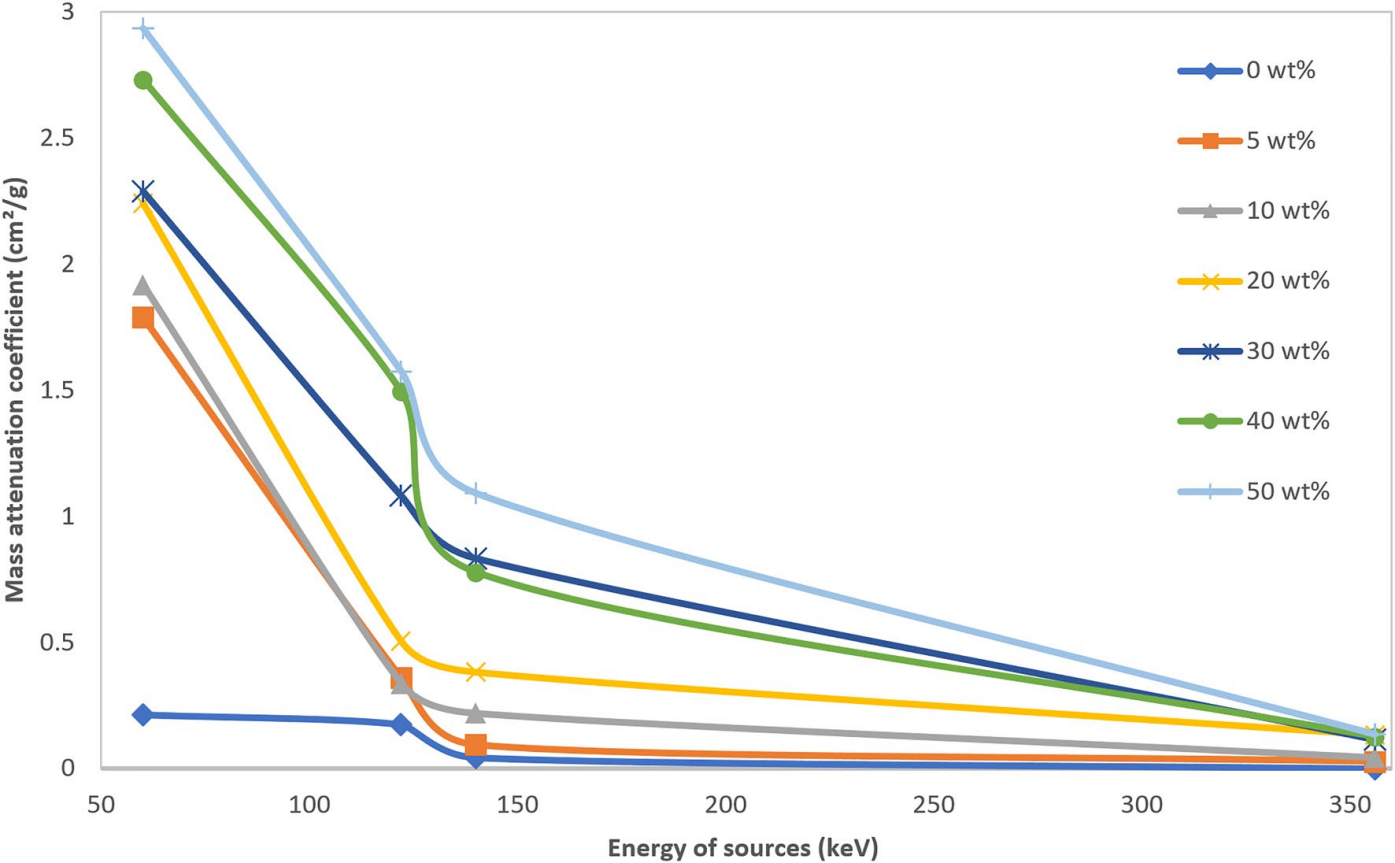
Mass attenuation coefficient of different wt% of PC-Bi_2_O_3_ nanocomposite at various energies.

When the gamma photons interact with the bounded electrons of Bi_2_O_3_ (bismuth atomic number: 83), the photoelectric effect is dominant for the low gamma energy. So a combination of the high atomic number of the host medium and low energy gamma rays leads to the highest attenuation. Thus for ^241^Am, ^57^Co, and ^99m^Tc, the most probable interaction is photoelectric, but for ^133^Ba, with medium gamma energy, Compton scattering probability will increase; therefore, as shown in Fig. 7, for the same weight fraction of nanoparticles, at higher energies, the mass attenuation coefficient is decreased.

**Figure 8:**
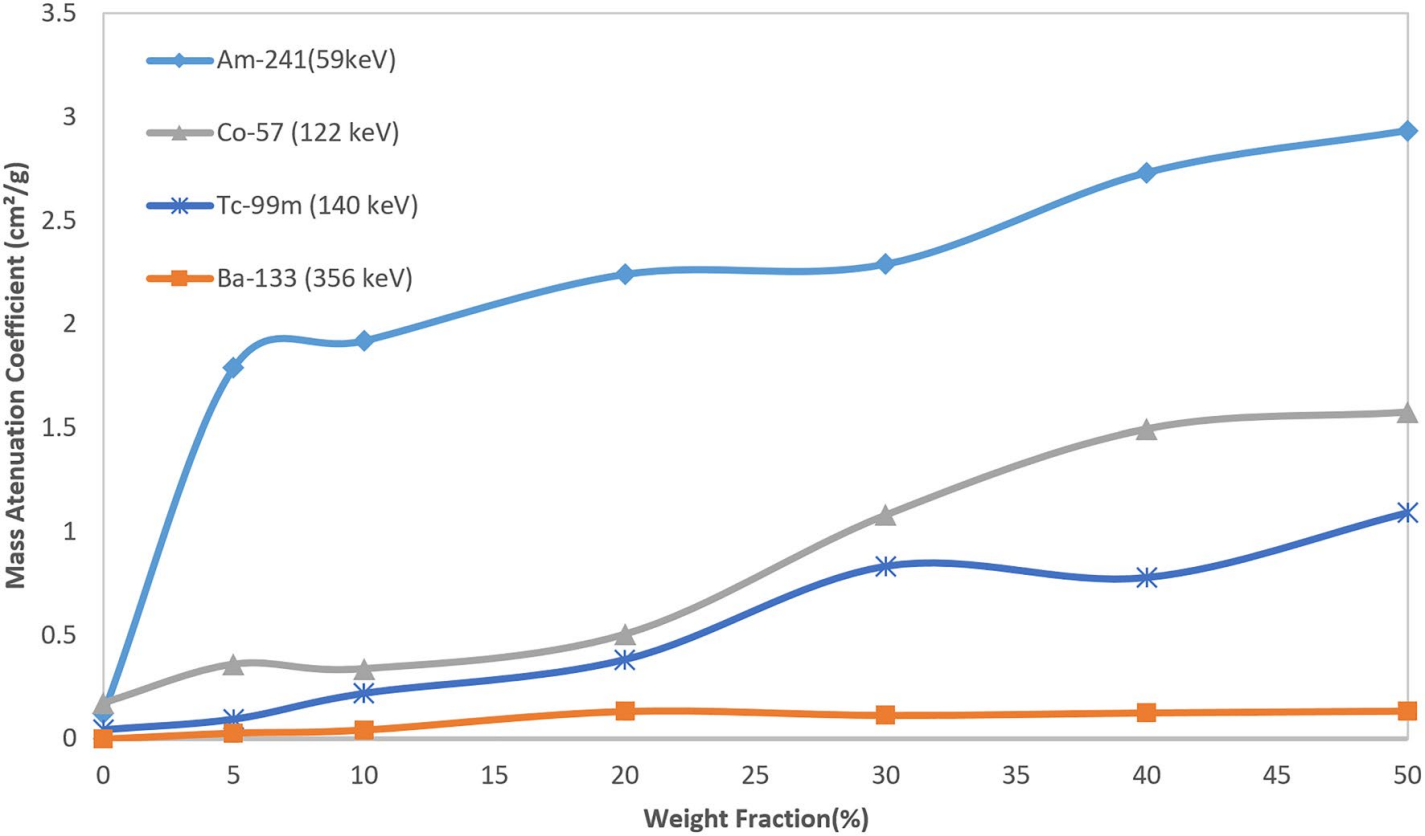
Mass attenuation coefficient result of different specimens for four gamma sources.

Comparing the results of four different gamma energies proved that increasing nano Bi_2_O_3_ wt% in the host polymer increases the ability to shield the gamma-rays, especially at low energies less than 140 keV. Noticing Fig. 8, it can be observed that for all sources, up to 30 wt%, the rate of increasing the amount of mass attenuation coefficient is fast. However, with the increasing concentration of nanoparticles into the polymer matrix, the rate of increasing µ/ρ tends to exhibit slower behavior. This happens due to the agglomeration nature of the nanomaterials, which leads to a non-homogeneous distribution in the polymer matrix. On the other hand, adding the reinforcement phase to the polymer matrix results in strengthen the structure, but overloading the reinforcement phase may lead to an unstable structure.

**Figure 9:**
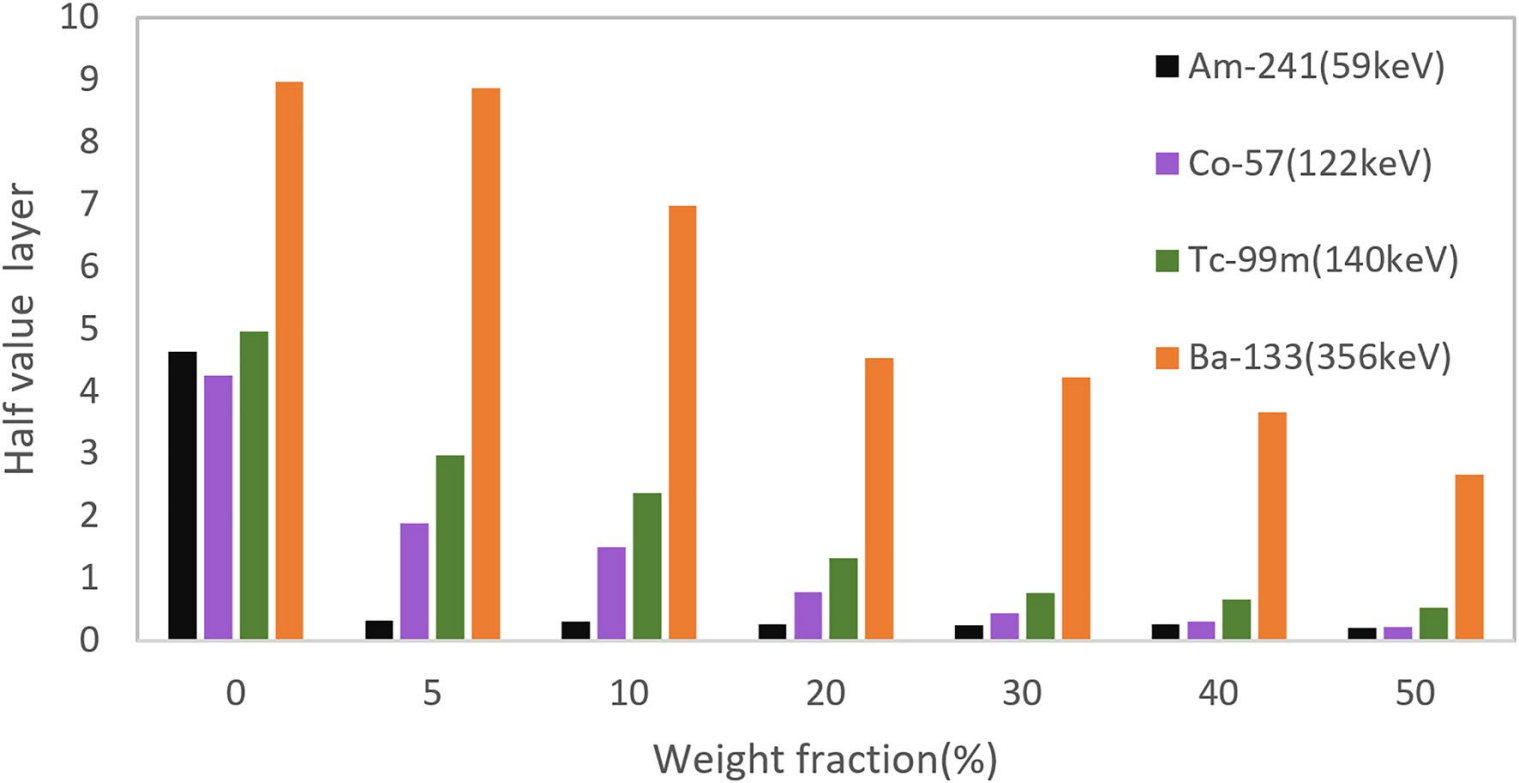
Half value layer results for different filler levels of PC/Bi_2_O_3_ nanocomposites.

**Figure 10:**
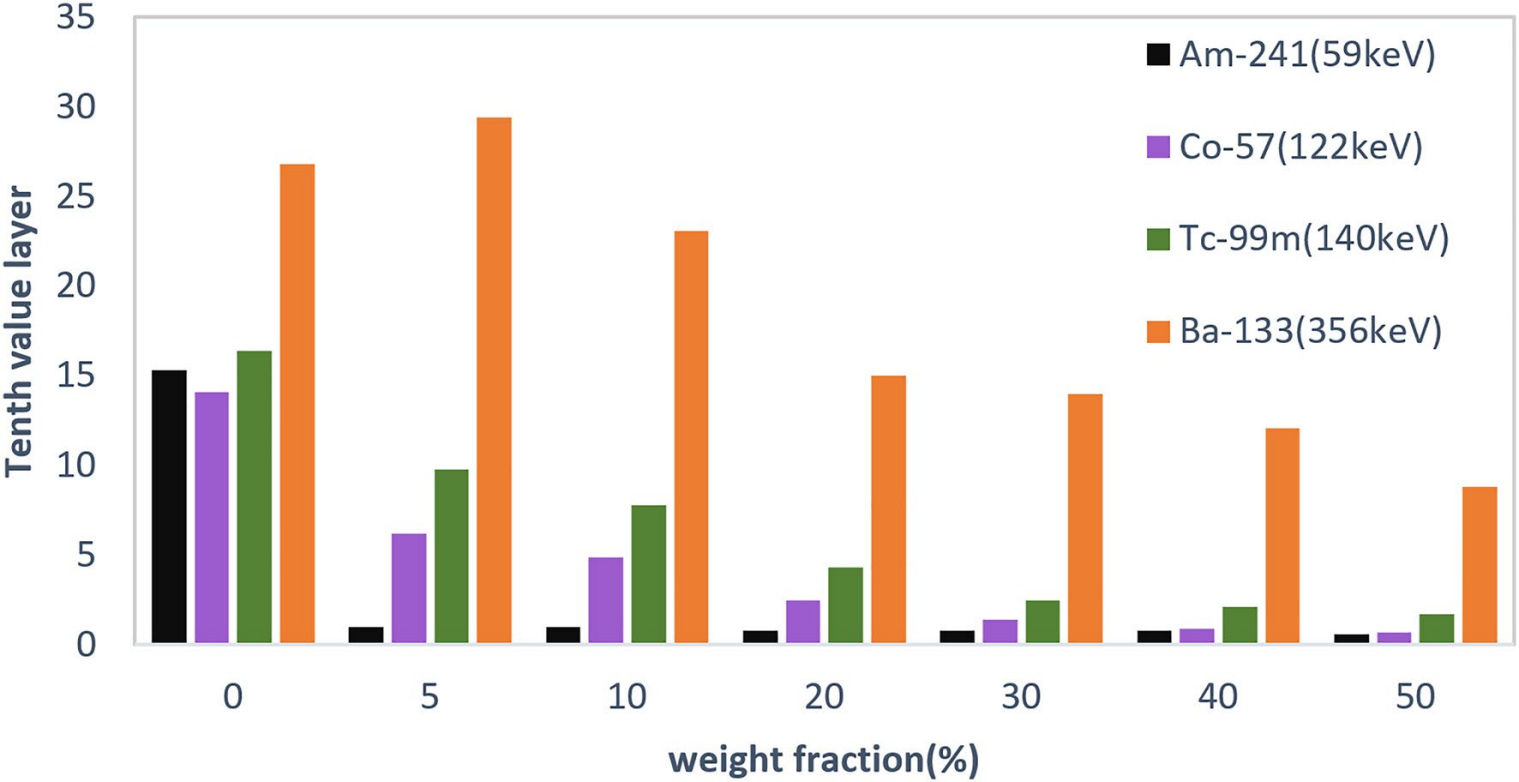
Tenth value layer results for different filler levels of PC/Bi_2_O_3_ nanocomposites.

As specified in Table 7, HVL and TVL results show that by adding only 5 wt% of filler level, the amount of HVL decreases significantly for low energy gamma-rays of 59 keV. The notable point is that for 356 keV gamma energy, no particular changes for the first HVL were observed until 20 wt%. As the gamma energy increases at a constant filler wt%, the amounts of HVL and TVL increase too, which is also shown in Figs. 9 and 10.

**Table 7:** HVL and TVL data for different filler levels of PC/Bi_2_O_3_ nanocomposites.

Gamma energy (keV)	Filler wt%						
	0	5	10	20	30	40	50
**59**							
HVL (cm)	4.6	0.3	0.3	0.2	0.2	0.2	0.1
TVL (cm)	15.3	1.0	1.0	0.8	0.8	0.8	0.6
**122**							
HVL (cm)	4.2	1.8	1.4	0.7	0.4	0.4	0.2
TVL (cm)	14.1	6.2	4.9	2.5	1.4	0.9	0.7
**140**							
HVL (cm)	4.9	2.9	2.3	1.3	0.7	0.6	0.5
TVL (cm)	16.4	9.8	7.8	4.3	2.5	2.1	1.7
**356**							
HVL (cm)	8.0	8.8	6.9	4.5	4.2	3.6	2.6
TVL (cm)	26.8	29.4	23.1	15.0	14.0	12.1	8.8

## Conclusion

In summary, gamma radiation shielding characteristics of Polycarbonate-Bismuth Oxide nanocomposite in various weight percentages of nanofillers of Bi_2_O_3,_ namely 0, 5, 10, 20, 30, 40, and 50 wt% were carried out. Mixed-solution method was used to fabricate the nanocomposite. SEM images showed a uniform dispersion of the inclusions into the polymer matrix. Also, XRD analysis was carried out and revealed the presence of the Bismuth nanoparticle in the composite. TGA and DTA analyses implied that as the nano-fillers content increased, the rate of weight loss of the nanocomposites decreased accordingly and the glass transition temperature of the nanocomposites shifted towards the lower temperatures.

In the following for each weight percentage of Bi_2_O_3_ in the composite, mass attenuation coefficient, HVL and TVL were measured. The measurements were repeated in the same way utilizing ^99m^Tc, ^241^Am, ^57^Co, and ^133^Ba point sources of gamma rays. Taking high photon absorption cross section of Bi_2_O_3_ nanoparticles into account, increasing weight percentage of the inclusions would cause a significant increase in mass attenuation coefficient. Results of the experiments proved that in different weight percentages, the enhancement of the concentration of the inclusions directly reduced the HVL and TVL values.

It can be concluded that at higher amounts of Bi_2_O_3_ wt% greater than 40 wt%, due to agglomeration, the value of mass attenuation coefficient in these nanocomposite tends to saturate, especially in the higher energies. Finally, results showed that PC-Bi_2_O_3_ nanocomposite as a lead-free material, exhibited convenient shielding characteristics, especially for low-energy gamma-rays, which could be a suitable substitute for traditional radiation shielding at the nuclear medicine level.
